# Detection of Abnormal Spontaneous Brain Activity Patterns in Patients With Orbital Fractures Using Fractional Amplitude of Low Frequency Fluctuation

**DOI:** 10.3389/fneur.2022.874158

**Published:** 2022-07-13

**Authors:** Min Kang, YuXuan Gao, LiJuan Zhang, RongBin Liang, QiuYu Li, HuiYe Shu, YiCong Pan, Ping Ying, SanHua Xu, Shao Yi

**Affiliations:** ^1^Department of Ophthalmology, The First Affiliated Hospital of Nanchang University, Jiangxi Branch of National Clinical Research Center for Ocular Disease, Nanchang, China; ^2^Department of Orthopaedics, The Second Affiliated Hospital of Shanxi Medical University, Taiyuan, China

**Keywords:** orbital fractures, fALFF, spontaneous brain activity, resting state, image

## Abstract

**Background:**

To date, no in-depth study has been conducted on the intrinsic pathological relationship between altered brain activity and related behavioral changes in patients with orbital fracture (OF).

**Purpose:**

The present research aimed to explore the potential functional network cerebrum activities in patients with OF using resting state functional magnetic resonance imaging–fractional amplitude of low-frequency fluctuation (rsfMRI-fALFF). This technique can reveal dynamic functional changes in specific cerebrum areas.

**Methods:**

Twenty patients with OF and 20 healthy controls (HCs) were included, closely matched in terms of gender, age, weight, and education level. To record spontaneous cerebral activity changes, the rsfMRI-fALFF tool was applied. Receiver operating characteristic (ROC) curves and Pearson's correlation analysis were used to analyze mean fALFF values in specific cerebrum regions and to explore changes of behavioral changes in patients with OF. The Hospital Depression and Anxiety scale was applied to reveal the relationship between emotional states and fALFF values of the right superior temporal gyrus in patients with OF.

**Results:**

In comparison with HCs, significantly lower fALFF values were detected in the left anterior cingulate gyrus (LACG) and right superior temporal gyrus (RSTG) in patients with OF. ROC curve analysis showed excellent accuracy. The mean fALFF values of the RSTG negatively correlated with the depression score as well as the anxiety score.

**Conclusion:**

The finding of abnormal spontaneous activities in cerebral regions may contribute to more comprehensive understanding of the potential neural network changes in patients with OF. The changes of fALFF value in patients with OF may help to gauge their emotional changes and clinical recovery levels.

## Introduction

The orbital bones are fragile, with no protective surround, making them vulnerable to orbital wall fracture (1), of which trauma is the main cause. Previous research (2) has shown that about half of orbital fractures are isolated and are usually limited to one orbital wall, the orbital floor, and medial wall being the most frequently occurring fracture sites (3). Orbital fractures mostly occur in children and young people, and are more common in males than females (4, 5). Chi et al. reviewed 733 cases of orbital fractures, among which three quarters were male (6). Conservative treatment is often used for small orbital fractures without displacement (7), while, for large displaced fractures, surgical intervention is necessary (8, 9). Orbital fractures may cause exophthalmos (10), enophthalmos (11), diplopia (12), entropion (13), subconjunctival hemorrhage (14), and even blindness (15). Therefore, early monitoring and termination of adverse disease progression in patients with OF are very important. The use of modern imaging technology to study the brain activity of patients with OF may be important as a means by which to improve understanding of the mechanism of potential pathological changes in this condition, and may, therefore, be beneficial to the management of complications. Previous studies have confirmed that the changes of spontaneous brain activity in related brain regions can be used as an indicator of disease progression. Therefore, we tried to explore the value of using modern imaging techniques to explore the value of spontaneous brain activity changes as a marker of disease progression in patients with orbital fractures.

Magnetic resonance imaging (MRI), as a widely used auxiliary imaging technology, was developed in the 1980s and provides us a preliminary understanding of the anatomical structure and operating mechanism of the brain (16, 17). Hemodynamic changes caused by neuronal activity can be qualitatively measured with the help of MRI technology, known as functional magnetic resonance imaging (fMRI). This approach has been used in a variety of studies on the mechanism and effects of spontaneous neuronal activity in the brain, and has been helpful in exploring the pathophysiological changes and pathogenesis of various diseases (18, 19). The fractional amplitude of low frequency fluctuation (fALFF), a resting state fMRI method, has provided an index for the evaluation of spontaneous neural activity, and its accuracy and sensitivity have been widely confirmed (16). To our knowledge, the present experiment was the first attempt to explore the connection between spontaneous brain activity and behavioral performance in patients with OF using the fALFF method as well as to explore the value of fALFF in evaluating the pathological changes and severity of OF.

## Subjects and Methods

### Subjects

In total, 20 patients with OF (12 males, 8 females) and 20 matched healthy controls (HCs) participated in this research. The relevant inclusion criteria were: (1) with optic nerve injury; (2) with diplopia; (3) with orbital collapse; (4) with limited eye movement; (5) with surgical treatment; (6) no other ocular diseases (such as macular degeneration); (7) no brain disease (such as cerebral infarction); (8) no history of mental illness; (9) no organic diseases likely to affect MRI examination.

The 20 HCs (12 males, 8 females) were highly similar to the OF group in sex, age, weight, and education level. Our study met the ethical standards of the Medical Ethics Committee of the First Affiliated Hospital of Nanchang University as well as the principles of the Declaration of Helsinki. After materials, methods, purpose, and underlying risks of this experiment were explained, each participant signed a declaration of informed consents.

### MRI Parameters

MRI scanning was conducted using a Trio 3-Tesla MR scanner (Trio; Siemens, Munich, Berlin, Germany) in all the participants. During the MRI scanning, other interference factors were excluded, and the subjects remained awake, breathing normally and with good vital signs. The whole-brainT1-weights were obtained with the application of the spoiled gradient-recalled echo sequence. Relevant corresponding parameter settings of structural images were as follows: echo time = 2.25 ms, repetition time = 1,800 ms, field of view = 250 × 250 mm^2^, layer interval = 0.5 mm, flip angle = 90°, matrix = 256 × 256, thickness = 1 mm. Functional images (*n* = 240) were captured with the following settings: echo time = 30 ms, repetition time = 2,000 ms, field of view = 220 × 220 mm^2^, flip angle = 90°, matrix = 64 × 64, thickness = 4 mm.

### fMRI Data Processing

All data were pre-filtered using MRIcro (www.MRIcro.com) and then preprocessed the filtered data using SPM8 (https://www.fil.ion.ucl.ac.uk/spm/). In pre-filtering, the first 10 volumes were regarded as invalid data and excluded to ensure steady signals. The volumes were offset by no more than 2 mm in X, Y, or Z directions. On the basis of the standard echo planar imaging template, the images were resampled and normalized (with a standard setting of voxel size 3 × 3 × 3 mm, and were smoothed) to enhance the signal-to-noise ratio. This method has been described in detail previously (20).

### fALFF Analysis

To calculate fALFF, a full-width Gaussian kernel (half maximum = 6 × 6 × 6 mm^3^) was used to smooth the remaining 230 images. Band-pass (0.01–0.08 Hz) filtering was used to control for movement artifacts and low frequency drift. A fast Fourier transform (FFT) algorithm was used to obtain the signal power spectrum, and fAFLL was calculated as the ratio of the amplitude at each value in the low frequency band (0.01–0.08 Hz) to full-band (0–0.25 Hz) power amplitude.

### Brain-Behavior Correlation Analysis

To look for any associations between brain activity and behavioral performance, brain regions of interest were determined based on fALFF values, and Pearson's correlation analysis was used to explore the linear relationship between activities in these regions and clinical manifestations.

### Statistical Analysis

Using SPSS software version 20.0 (IBM Corp, Armonk, NY, USA), an independent sample *t*-test was conducted on the common clinical variables and demographic data of patients with OF and HCs using a 5% significance level. A two-sample *t*-test was used to compare the functional data. Based on Gaussian random field theory, the statistical threshold of the voxel level in multiple comparisons was set at *p* < 0.05. Gaussian random field theory was used to determine the significance of the functional image at the 5% level with a cluster size > 40 voxels. Using the mean fALFF in various cerebral regions of HCs and patients with OF, the areas under the ROC curves (AUC) were obtained. In addition, Pearson correlation analysis was used to look for associations between the mean fALFF values in multiple cerebrum regions and characteristics of clinical behavior in patients with OF.

## Results

### Demographics and Visual Measurements

No significant differences were found between groups in terms of gender (*p* > 0.99), weight (*p* = 0.902), or age (OF, 51.21 ± 11.42; HC, 50.96 ± 10.82; *p* = 0.871). However, best corrected monocular visual parameters were significantly different between groups, as follows: visual acuities (*p* = 0.017, left, and 0.011, right eye), visual-evoked potential (VEP) latencies (*p* = 0.022, left, and 0.017, right eye), and amplitudes (*p* = 0.012, left, and 0.009, right eye) (Table 1).

**Table 1:** Basic information of the participants in the study.

**Condition**	**OF**	**HCs**	**t**	***P*-value**
Male/female	12/8	12/8	N/A	>0.99
Age (years)	51.21 ± 11.42	50.96 ± 10.82	0.242	0.871
Weight (kg)	68.32 ± 9.24	69.93 ± 9.54	0.165	0.902
Handedness	20R	20R	N/A	>0.99
Duration of (days)	11.61 ± 4.14	N/A	N/A	N/A
Best-corrected VA-left eye	0.40 ± 0.20	1.05 ± 0.20	−3.763	0.017
Best-corrected VA-right eye	0.45 ± 0.15	1.00 ± 0.15	−3.064	0.011
Latency (ms)-right of the VEP	118.16 ± 8.29	100.98 ± 6.17	3.554	0.017
Amplitudes(uv)-rightof the VEP	6.87 ± 2.42	14.16 ± 1.93	−6.643	0.009
Latency (ms)-left of the VEP	116.12 ± 7.11	101.21 ± 1.32	4.532	0.022
Amplitudes (uv)-left of the VEP	7.42 ± 2.73	16.74 ± 2.52	−5.732	0.012

*Compare two groups with independent t-tests (p < 0.05). VA, visual acuity; N/A, not applicable; OF, orbital fractures; VEP, visual-evoked potential; HCs, healthy controls*.

### Differences in fALFF

**Figure 1:**
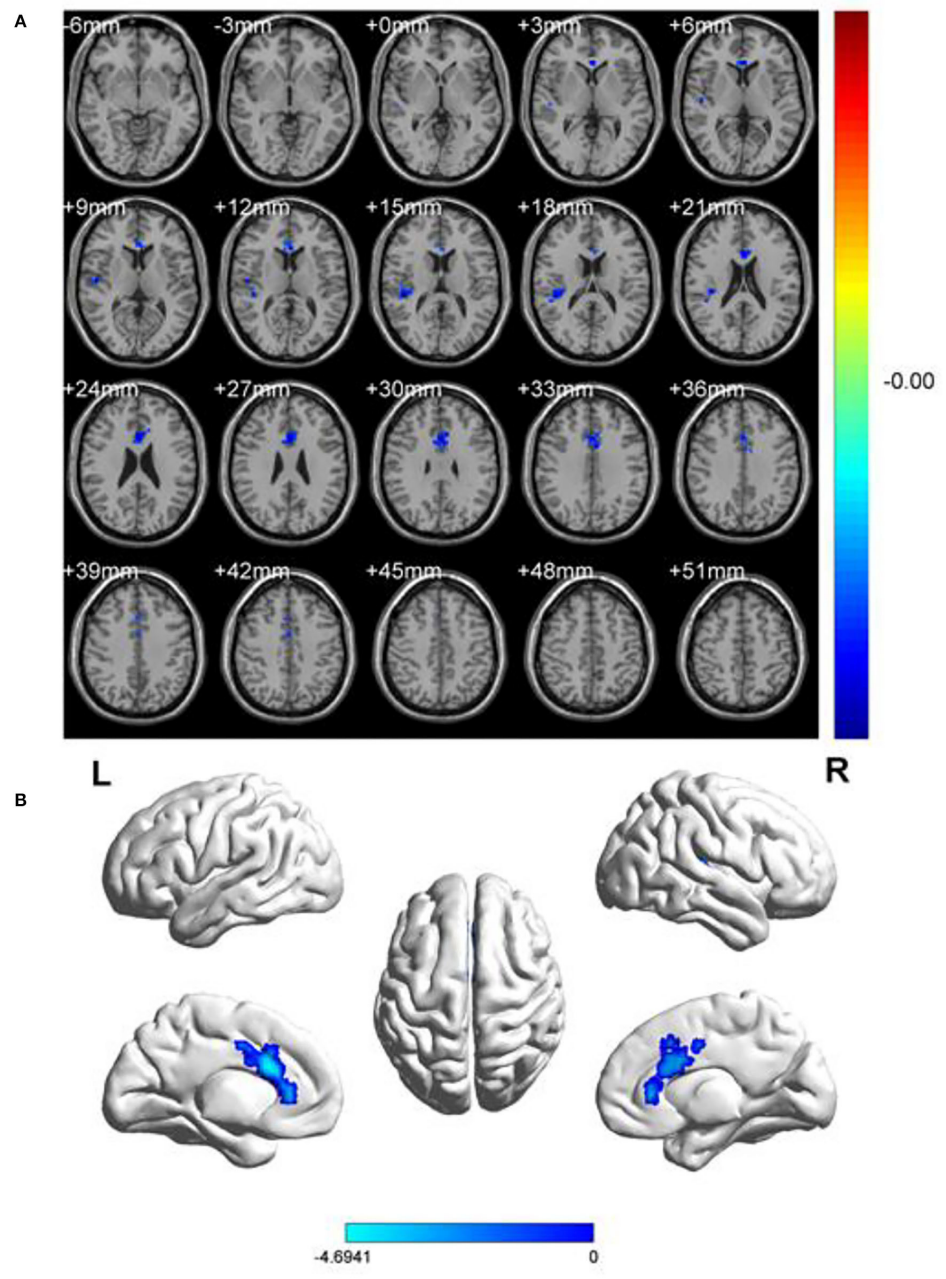
Spontaneous brain activities of and healthy controls. **(A)** Different fALFF areas in patients with OF. **(B)** The blue areas represented lower fALFF values. L, left; R, right.

**Figure 2:**
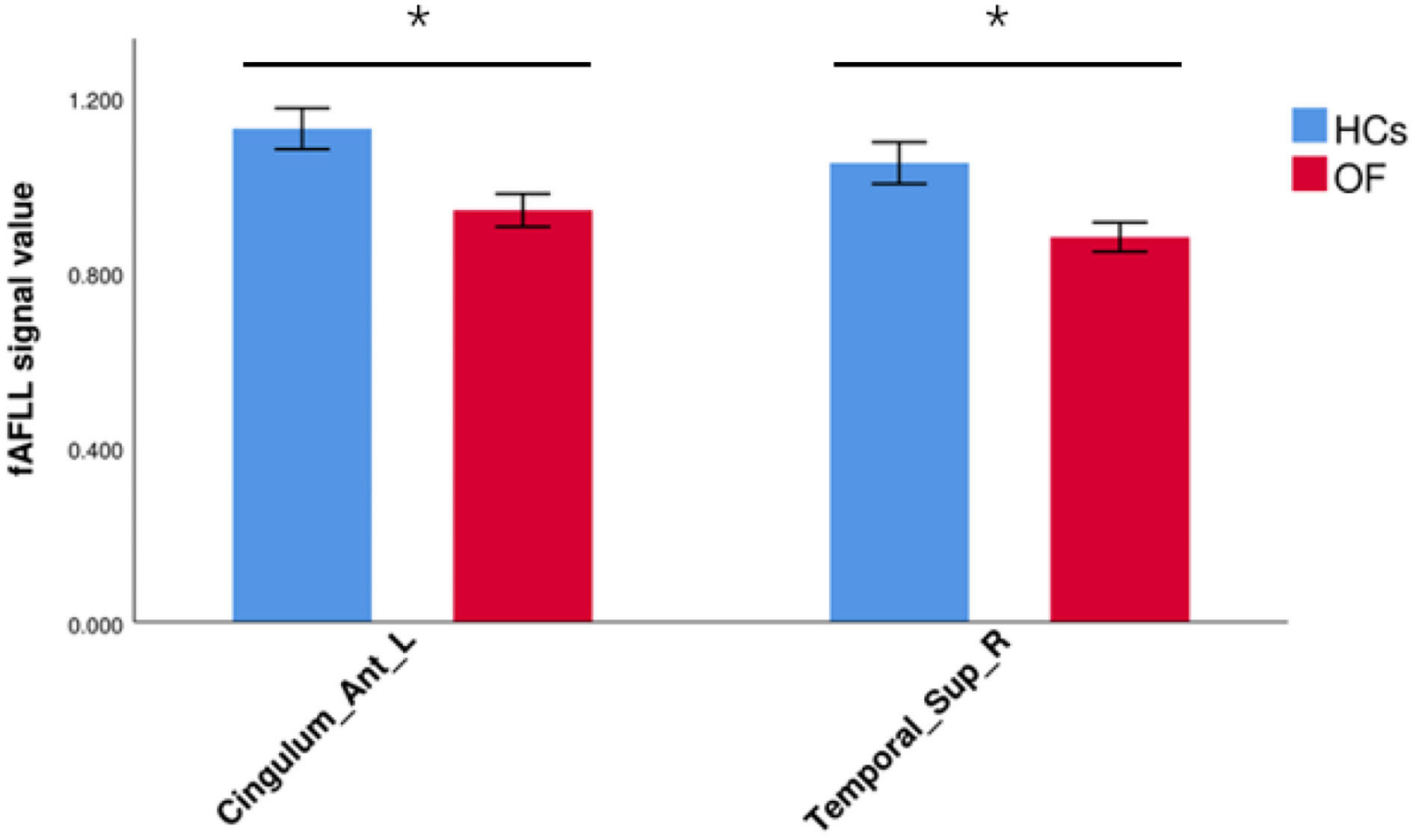
The average fALFF values in OF and HC groups. Cingulum_Ant_L, left anterior cingulate gyrus; Temporal_Sup_R, right superior temporal gyrus; fALFF, fractional amplitude of low-frequency fluctuation; OF, orbital fractures; HC, healthy control. “*” *p* < 0.05.

In comparison with HCs, the patients with OF showed significant lower fALFF values in the left anterior cingulate gyrus (LACG) and right superior temporal gyrus (RSTG) (Figure 1, Table 2). The mean fAFLL values are shown in Figure 2.

**Table 2:** Brain areas with significant differences in fALFF between two groups.

**Brain areas**	**MNI coordinates**			**BA**	**Number of voxels**	***T* value**
	**X**	**Y**	**Z**			
Patient < HC						
Cingulum_Ant_L	0	18	27	31	172	−4.6941
Temporal_Sup_R	45	−30	15	82	82	−4.399

*A P-value < 0.05 was significantly different for multiple comparisons using Gaussian random field theory (cluster 0.40 voxels, Alphasim corrected). HC, health control; MNI, Montreal Neurological Institute; BA, Brodmann area; Cingulum_Ant_L, left anterior cingulate gyrus; Temporal_Sup_R, right superior temporal gyrus*.

### Receiver Operating Characteristic Curves

**Figure 3:**
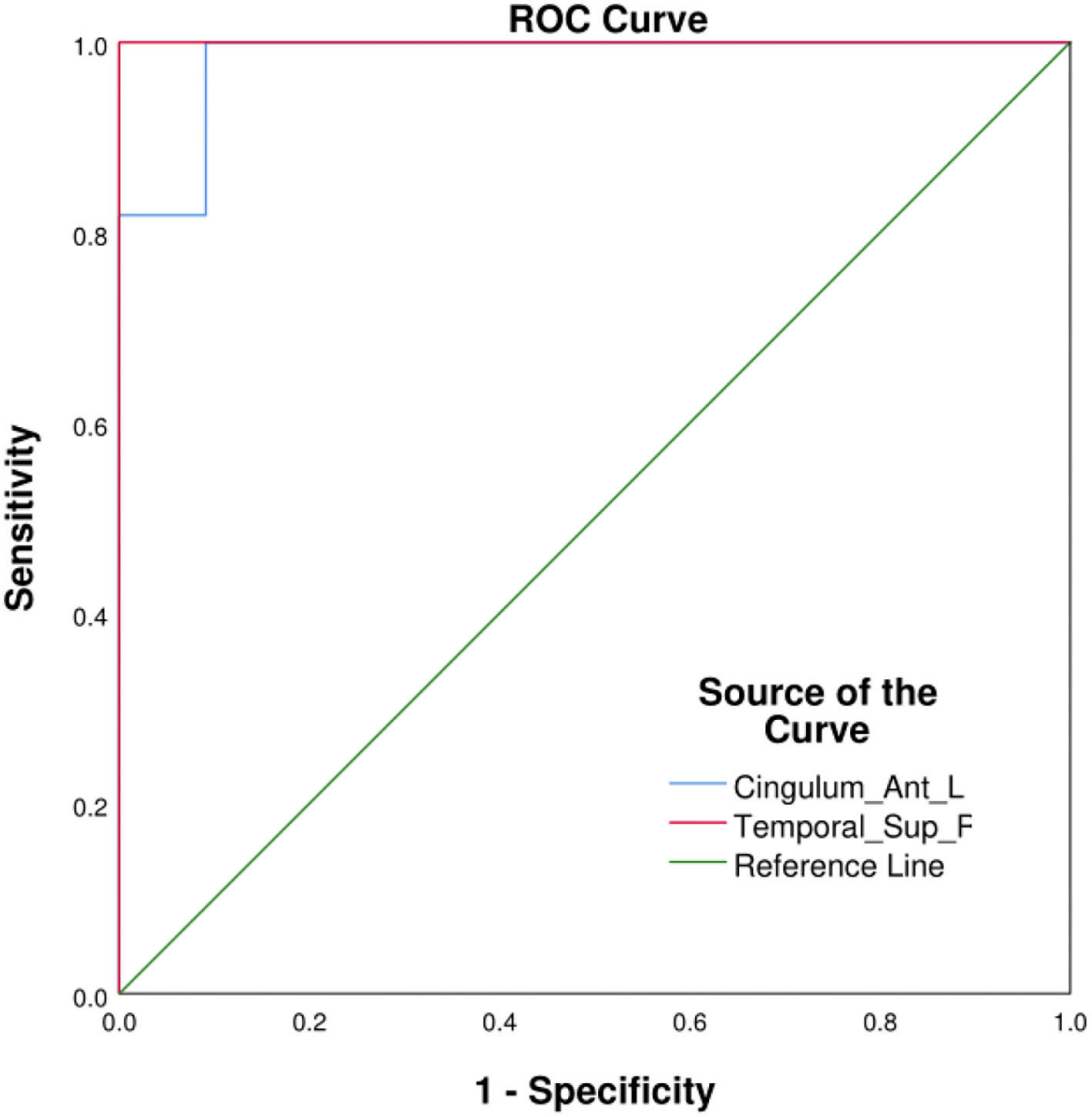
ROC curve analysis of the average fALFF values in different cerebrum areas. AUCs: Cingulum_Ant_L: 0.983, Temporal_Sup_R: 1.000. Cingulum_Ant_L, left anterior cingulate gyrus; Temporal_Sup_R, right superior temporal gyrus; AUC, area under the curve; ROC, receiver operating characteristic.

ROC curves were used to visualize the comparison between average fALFF values of patients with OF and HCs, and the areas under the curves (AUCs) were used as indicators of diagnostic accuracy. Using this approach, AUCs of the LATG and RSTG were found to be 0.983 and 1.000, respectively (Figure 3).

### Correlation Analysis

**Figure 4:**
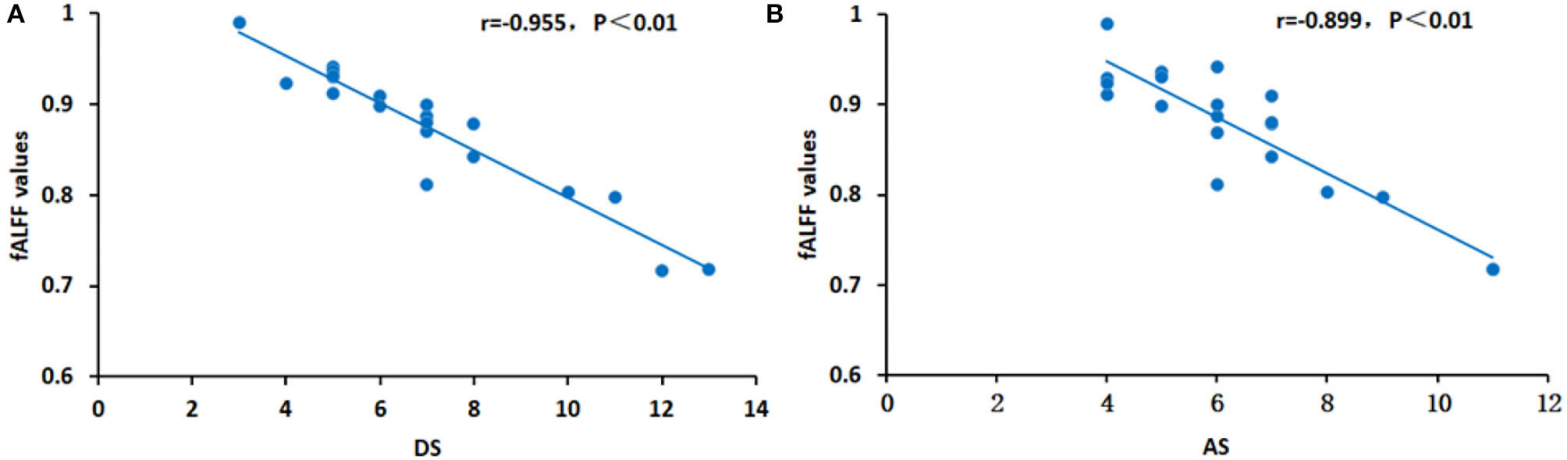
Correlations between the average fALFF values and clinical characteristics in the RSTG. In the RSTG, the DS (r = −0.955, *p* < 0.01) **(A)** and AS (r = −0.899, *p* < 0.01) **(B)** are both represented by a negative relationship with the fALFF values. fALFF, fractional amplitude of low-frequency fluctuation; DS, depression score; AS, anxiety score; RSTG, right superior temporal gyrus.

In patients with OF, significant correlations were found between fALFF values in the RSTG and depression scores (negative correlation: r = −0.955, *p* < 0.01) and anxiety scores (negative correlation: r = −0.899, *p* < 0.01) (Figure 4).

## Discussion

**Figure 5:**
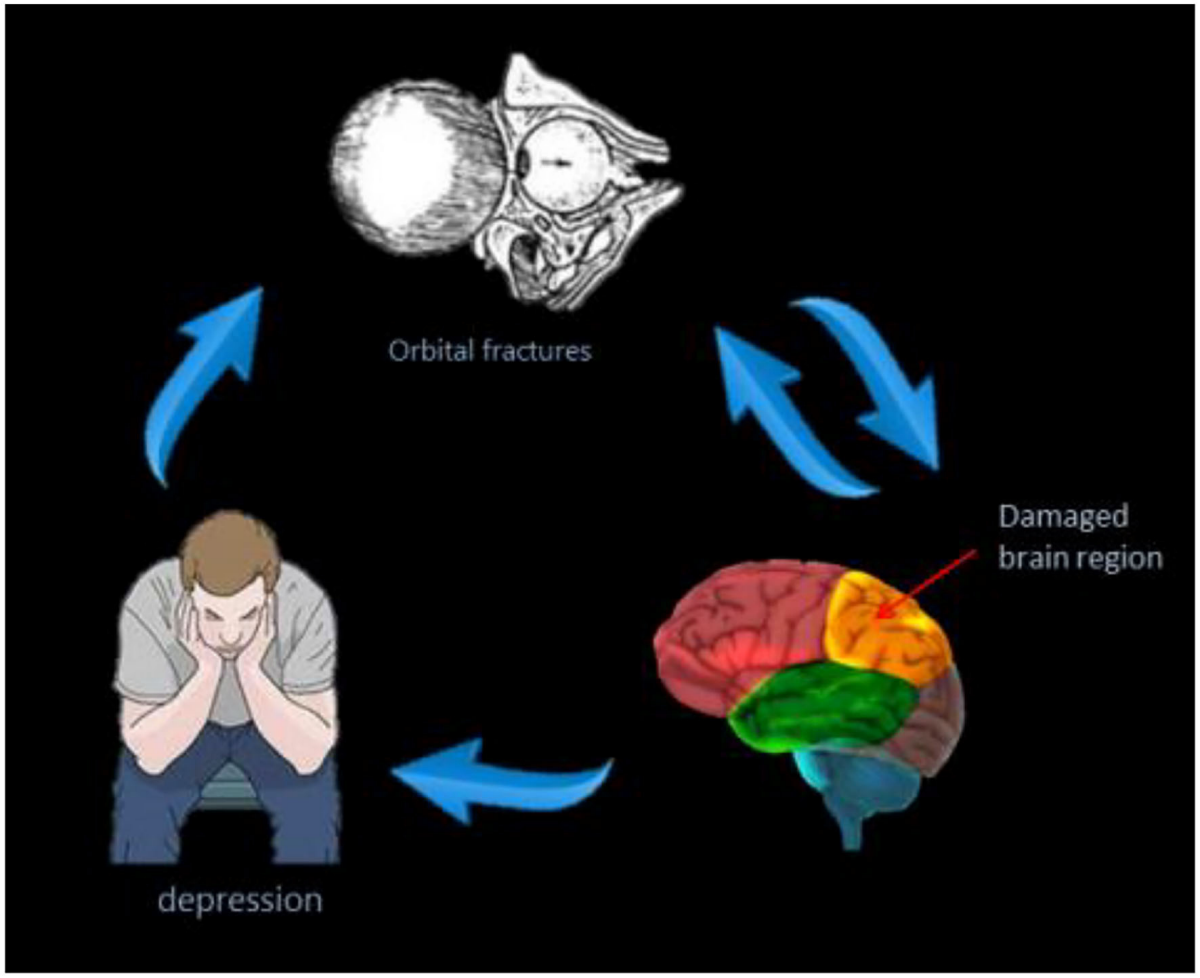
The correlations between average fALFF signal values and clinical manifestation of patients with OF. The patients with OF have lower fALFF values, and they are more likely to develop depressive symptoms. OF, orbital fractures.

**Figure 6:**
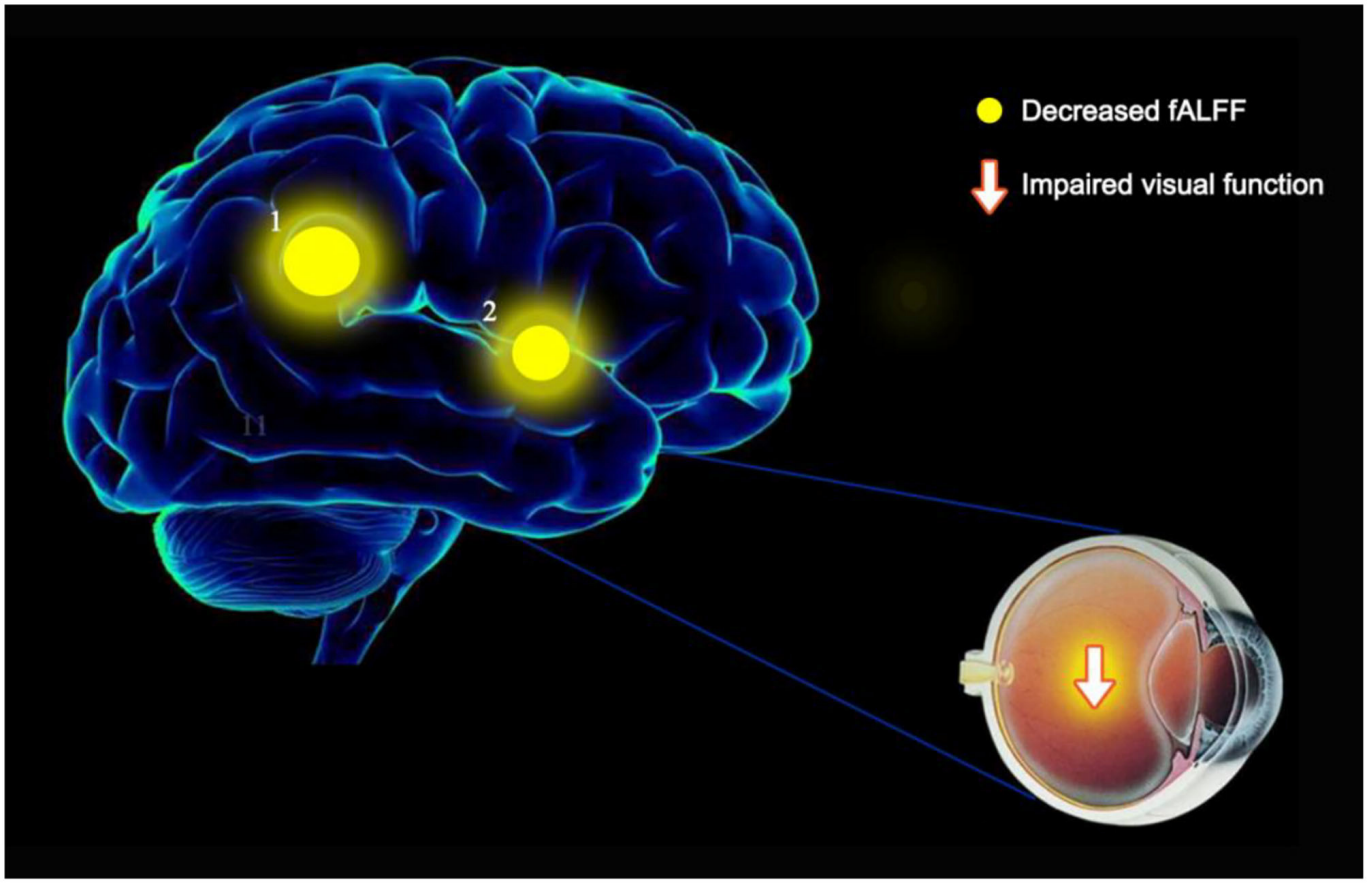
fALFF results of cerebral activity in the OF group. The fALFF values of the cerebral areas in the OF group were in the following: 1- left anterior cingulate gyrus (t = −4.6941), 2 - right superior temporal gyrus (t = −4.399). The intensity, as well as the frequency of brain activity, is reflected by the size of spots.

To our knowledge, the ALFF method has not previously been used to study the potential relationship between brain activity changes and clinical manifestations in patients with OF. This study aimed to explore the cerebral neural changes after orbital fracture using the fALFF technique (Figure 5). The study found significantly lower fALFF values in the LACG and the RSTG in patients with OF (Figure 6). In previous studies, the fALFF method has been applied to a series of ophthalmological diseases, including normal-tension glaucoma (20), monocular blindness (21), retinal vein occlusion (22), diabetic retinopathy, and nephropathy (23) (Table 3), demonstrating its potential for clinical application.

**Table 3:** The fALFF method applied in ophthalmological diseases.

**Author**	**Year**	**Disease**	**Brain areas**	
			**Increased fALFF values**	**Decreased fALFF values**
Li et al. (20)	2020	Normal-tension glaucoma	_	RAG, RACL
Fang et al. (21)	2020	Monocular blindness	LP, RPI, LPI	LCA
Tong et al. (22)	2020	Retinal vein occlusion	LC, RC, RB, LI	RC, RT
Wang et al. (23)	2021	Primary angle-closure glaucoma	BSFG	LC, LMTG RMTG, RPG

*fALFF, fractional amplitude of low-frequency fluctuation; RAG, right angular gyrus; LP, left precuneus; LMTG, left middle temporal gyrus; LCA, left anterior cingulate; RPI, right inferior parietal lobe; LPI, left inferior parietal lobe; RMTG, right middle temporal gyrus; LC, left cerebellum; RC, right cerebellum; RB, right brainstem; RC, right calcarinesulcus; BSFG, bilateral superior frontal gyrus; LIG, left lingual gyrus; RPG, right precentral gyrus; RT, right thalamus; LI, left insula; RACL, right anterior cuneiform lobe*.

The anterior cingulate gyrus (ACG) is a functional area associated with many physiological functions, is located in the medial brain and passes longitudinally through the parietal lobe, and its main roles are in memory (24), action – outcome learning (25–27), emotion, and reward-related processing (28). The research of Hornak et al. (29) showed that, in some cases, the ACG plays an important part in voice and facial expression recognition, while Lane et al. (30–32) studied anterior cingulate injury in subjective emotional experience, and found that ventral ACG and Brodmann's area 9 may be activated during mood fluctuations. Based on the functions of the anterior cingulate gyrus, some researchers have explored its diagnostic value in Parkinson's disease (33), depression (34), and acute and chronic pain (35). In addition, a previous study has found that the prefrontal cingulate gyrus can respond to visual stimuli (36). In the present study, given the reduced visual responses in patients with OF, the results may indicate a compensatory mechanism for vision loss in patients with OF.

The superior temporal gyrus (STG) is a functional area of the brain located in the temporal lobe, closely related to emotional and speech processing (37, 38). The STG is a component of the default mode network, which is inhibited during brain activity and excited during rest. Liu et al. (39) used the rsMRI-fALFF method to study the STG in depression. They found that the lower fALFF values of STG correlated greater reductions on the Hamilton rating scale for depression, and inferred that STG neural changes were closely related to the effect of early treatment for depression. In addition, Wang et al. (40) measured functional connectivity density in STG and found that abnormal connectivity is negatively correlated with the treatment effect. In the present experiment, the fALFF value of the right STG in patients with OF was significantly lower than that in healthy controls, and we speculate that this decrease may be a compensatory mechanism for the recovery of brain function in patients with OF. The results suggest that the fALFF value may be used as a reliable index to gauge therapeutic effects of clinical treatment. Moreover, it was discovered that, in the patients with OF, fALFF values in the RSTG were negatively correlated with anxiety and depression scores, which may indicate a self-regulation mechanism in this brain area, with brain function being temporarily inhibited (Table 4).

**Table 4:** Brain areas of altered fALFF values and anticipated results.

**Brain regions**	**Experimental result**	**Brain function**	**Anticipated results**
Cingulum_Ant_L	OF < HCs	Memory, action–outcome learning, emotion and reward-related processing	Behavioral disorders, memory impairment, depression,
Temporal_Sup_R	OF < HCs	Emotional and speech processing, curative effect index	Mental disorders, speech disorder, reflecting treatment effect

*Cingulum_Ant_L, left anterior cingulate gyrus; Temporal_Sup_R, right superior temporal gyrus; OF, orbital fractures; HCs, healthy controls*.

This study has some limitations; one of which is the relatively small sample size, and the other is that the sample source was limited and not completely matched. Third, compared with the simple use of VEP, the use of pattern electro retino grams (PERGs) and pattern visual-evoked potentials (PVEPs) two checks can be more rigorous explanation of the problem. Therefore, future research should use larger and more closely matched samples to further clarify the neural changes in patients with orbital fractures, and to provide a more intuitive clinical efficacy index for treatment. In conclusion, this study has demonstrated that patients with OF have reduced fALFF values in specific cerebrum areas, indicating changes in spontaneous brain activity. Further research on the mechanism underpinning brain activity changes in patients with OF may be helpful to advance understanding of this condition.

## Data Availability Statement

The original contributions presented in the study are included in the article/supplementary material, further inquiries can be directed to the corresponding author/s.

## Ethics Statement

The studies involving human participants were reviewed and approved by Ethics Committee of Nanchang University. The patients/participants provided their written informed consent to participate in this study.

## Author Contributions

YXG, SHX, PY, YCP, HYS, LJZ, RBL, and QYL: Data collation, data analysis, and paper writing and revision. SY: The funding and design of the project and the guidance of the article. All authors contributed to the article and approved the submitted version.

## Conflict of Interest

The authors declare that the research was conducted in the absence of any commercial or financial relationships that could be construed as a potential conflict of interest.

## Publisher's Note

All claims expressed in this article are solely those of the authors and do not necessarily represent those of their affiliated organizations, or those of the publisher, the editors and the reviewers. Any product that may be evaluated in this article, or claim that may be made by its manufacturer, is not guaranteed or endorsed by the publisher.
